# Fabrication of Eco-Friendly Polyelectrolyte Membranes Based on Sulfonate Grafted Sodium Alginate for Drug Delivery, Toxic Metal Ion Removal and Fuel Cell Applications

**DOI:** 10.3390/polym13193293

**Published:** 2021-09-27

**Authors:** Raagala Vijitha, Kasula Nagaraja, Marlia M. Hanafiah, Kummara Madhusudana Rao, Katta Venkateswarlu, Sivarama Krishna Lakkaboyana, Kummari S. V. Krishna Rao

**Affiliations:** 1 Polymer Biomaterial Design and Synthesis Laboratory, Department of Chemistry, Yogi Vemana University, Kadapa 516005, Andhra Pradesh, India; vijitha.ragaala@gmail.com (R.V.); nagarajakasula33@gmail.com (K.N.); 2 Department of Earth Sciences and Environment, Faculty of Science and Technology, Universiti Kebangsaan Malaysia, Bangi 43600, Selangor, Malaysia; mhmarlia@ukm.edu.my; 3 Centre for Tropical Climate Change System, Institute of Climate Change, Universiti Kebangsaan Malaysia, Bangi 43600, Selangor, Malaysia; 4 School of Chemical Engineering, Yeungnam University, 280 Daehak-Ro, Gyeongsan-si 38541, Gyeongsangbuk-do, Korea; 5 Laboratory for Synthetic & Natural Products Chemistry, Department of Chemistry, Yogi Vemana University, Kadapa 516005, Andhra Pradesh, India; kvenkat@yogivemanauniversity.ac.in; 6 Department of Chemical Technology, Chulalongkorn University, Pathumwan, Bangkok 10330, Thailand; svurams@gmail.com

**Keywords:** polyelectrolyte membrane, sodium alginate, graft copolymer, PVA, drug delivery, copper ion removal, fuel cell

## Abstract

Polyelectrolyte membranes (PEMs) are a novel type of material that is in high demand in health, energy and environmental sectors. If environmentally benign materials are created with biodegradable ones, PEMs can evolve into practical technology. In this work, we have fabricated environmentally safe and economic PEMs based on sulfonate grafted sodium alginate (SA) and poly(vinyl alcohol) (PVA). In the first step, 2-acrylamido-2-methyl-1-propanesulphonic acid (AMPS) and sodium 4-vinylbenzene sulfonate (SVBS) are grafted on to SA by utilizing the simple free radical polymerization technique. Graft copolymers (SA-g-AMPS and SA-g-SVBS) were characterized by ^1^H NMR, FTIR, XRD and DSC. In the second step, sulfonated SA was successfully blended with PVA to fabricate PEMs for the in vitro controlled release of 5-fluorouracil (anti-cancer drug) at pH 1.2 and 7.4 and to remove copper (II) ions from aqueous media. Moreover, phosphomolybdic acids (PMAs) incorporated with composite PEMs were developed to evaluate fuel cell characteristics, i.e., ion exchange capacity, oxidative stability, proton conductivity and methanol permeability. Fabricated PEMs are characterized by the FTIR, ATR-FTIR, XRD, SEM and EDAX. PMA was incorporated. PEMs demonstrated maximum encapsulation efficiency of 5FU, i.e., 78 ± 2.3%, and released the drug maximum in pH 7.4 buffer. The maximum Cu(II) removal was observed at 188.91 and 181.22 mg.g^–1^. PMA incorporated with PEMs exhibited significant proton conductivity (59.23 and 45.66 mS/cm) and low methanol permeability (2.19 and 2.04 × 10^−6^ cm^2^/s).

## 1. Introduction

Polyelectrolyte membranes (PEMs) gained considerable attention for their health, environment and energy applications due to their promising characteristics such as multi functionality, easy fabrication, low cost, biocompatibility, biodegradability and tunable physico-chemical properties [1,2,3,4,5]. Recently, polymer blends and their composites attracted much attention as PEMs owing to their potential properties as a carrier, separator and transporter in the fields of drug delivery [2,3], pervaporation/toxic metal ion removal and fuel cell [4,5], respectively. Hence, biodegradable and biocompatible (natural and synthetic) polymer derivatives have significant roles in designing and developing PEMs. The fluorinated polymer, Nafion^TM^, is a widely used PEM for a variety of applications, such as in fuel cells [6], microbial fuel cell [7], drug delivery [8], adsorption [9] and sensors [10], owing to potential electrochemical and mechanical properties of Nafion^TM^. However, it has some drawbacks such as hydrophobicity, high cost, difficulty in processing the material, non-biocompatibility, reduction in the material tissue interactions and high methanol permeability [11,12]. Hence, it is necessary to design multi-purpose polymer membrane with cost effectiveness in mind while ensuring environment protection.

Sodium alginate (SA) is a hydrophilic anionic polysaccharide extracted from the brown algae, which belongs to the family of *Laminariaceae*. The major sources are algae *Laminaria hyperborea*, *Macrocystis pyrifera* and *Ascophyllum nodosum* [13]. SA is a branched polymer, and its chemical composition consists of β-(1→4)-linked-D-mannuronic acid and α-(1→4)-linked L-guluronic acid residues. SA is biodegradable, biocompatible, less-toxic, good gelling and a stabilizing agent. Hence, it is used for medicinal purposes, food processing, cosmetics [14,15], pharmaceutical purposes, bioadhesive microsphere purposes, microencapsulation and drug delivery; and tissue engineering, biosensors, water saving material for agricultural and horticultural, adsorption of methylene blue, electrical devices, nanoparticles, microencapsulation and wound healing applications [16,17]. However, SA alone has some drawbacks such as SA membrane exhibiting high swelling and reduced mechanical property. 

In recent years, polysaccharide-based materials such as graft copolymers, blends and composites are widely used as PEMs for various potential applications such as drug delivery, extraction of precious metals, separation of liquid mixtures and fuel cells; this is due to their excellent properties such as strong electrolyte groups, biocompatibility, biodegradability and controllable swelling character [3,4,5,10,11,18]. Krishna Rao et al. synthesized polyelectrolyte complexes from chitosan-g-acrylamidoglycolic acid and hydroxyapatite for bone cement applications [19]. Liwei Jin et al. developed SA and chitosan-based polyelectrolyte films for the controlled release of theophylline [20]. Drug release is extended up to 25 h; these films are helpful for the controlled release of water-soluble drugs. Chalitangkoon et al. fabricated a silver loaded composite film from hydroxyethylacryl chitosan and SA [21]. These films are utilized for the application of wound healing as well as drug delivery. Sarwar et al. designed a biocompatible film from SA and poly(ethylene glycol)monovinyl ethers for the controlled release of ciprofloxacin hydrochloride. Most of the drug (~80%) is released within 120 min [22].

Industrial effluents such as heavy metals and organic dyes/pigments pollute the environment on a daily basis, however, in order to safeguard the environment, various treatment processes have been developed [23,24,25,26,27,28]. The removal of heavy metal ions from wastewater by using biopolymer-derived polymeric membranes is of current interest. Copper is an essential nutrient with a limit of less than 2 mg/L, and exceeding this limit is dangerous to the liver, gastrointestinal tract and nervous system [29,30]. Having functional group (-OH, -COO^−^, -CONH, SO_3_^−^, -NH_2_ and -C-O-C-) polymeric membranes can be used for the removal of toxic metals and organic pollutant from waste water. PEMs have been fabricated either by blending biopolymers or incorporating inorganic fillers into the biopolymer matrix [4,23,24,25,26,27,31]. To remove copper from aqueous media, various materials have been developed, i.e., PEG functionalized chitosan and poly(vinyl alcohol), PVA, blend membranes [32], PVA/SiO_2_ composite [33] and poly(vinyl pyrrolidone)/Fe_2_O_3_-Al_2_O_3_ nanocomposites [34].

Polyelectrolyte membranes are a crucial component of fuel cell technologies; nevertheless, they must exhibit significant proton conductivity at low temperatures as well as good physico-mechanical and thermal stability [35,36,37]. Several research papers have been published on the construction of polysaccharide-based PEMs for fuel cell applications [38,39,40,41]. However, when compared to pure polymer membranes, inorganic fillers (for example, silicotungstic acid, phosphotungstic acid and phosphomolybdic acid) incorporated with polymer membranes have exhibited improved proton conductivity of fuel cell membranes [42,43]. 2-Acrylamido-2-methyl-1-propanesulphonic acid (AMPS) and sodium 4-vinylbenzene sulfonate (SVBS) are the most commonly used acrylic monomers for the preparation of water-soluble polymers/copolymers/graft copolymers by free radical polymerization; hence, they are used widely for the development of PEMs. Free radical polymerization has several advantages, such as simple reaction setup, inexpensive green solvents and easy purification of the product, and it requires moderate conditions compared to other graft polymerization techniques such as γ-radiation, Fenton’s reagent, photolysis and thermolysis [44]. In the present investigation, to improve flexibility and physico-mechanical characteristics of PEM, SA is grafted with AMPS/SVBS in the first step, and SA is blended with PVA and crosslinked with glutaraldehyde in the second step. Furthermore, to accelerate the fuel cell characteristics of membrane, PEMs are doped with the phosphomolybdic acid, a hetero poly acid.

## 2. Experimental

### 2.1. Materials

2-Acrylamide-2-methyl-1-propanesulphonic acid (AMPS), sodium 4-vinylbenzene sulfonate (SVBS), poly(vinyl alcohol), (PVA, MWt: 75,000), phosphomolybdic acid (PMA) and 5-fluorouracil (5FU) were obtained from Sigma-Aldrich, Missouri, USA. Sodium alginate, (SA, MWt: Medium), potassium persulfate, ceric ammonium nitrate, glutaraldehyde (GA), hydrochloric acid, acetone and methanol were obtained from Merck, Mumbai India. The SA-g-AMPS and SA-g-SVBS graft copolymers were synthesized as per our previous work [45]. Extra pure cupric nitrate trihydrate was obtained from the FINAR chemicals, Ahmedabad, India. All experiments were carried out by using double distilled water.

### 2.2. Fabrication of Polyelectrolyte Membranes (PEMs) 

**Scheme 1:**
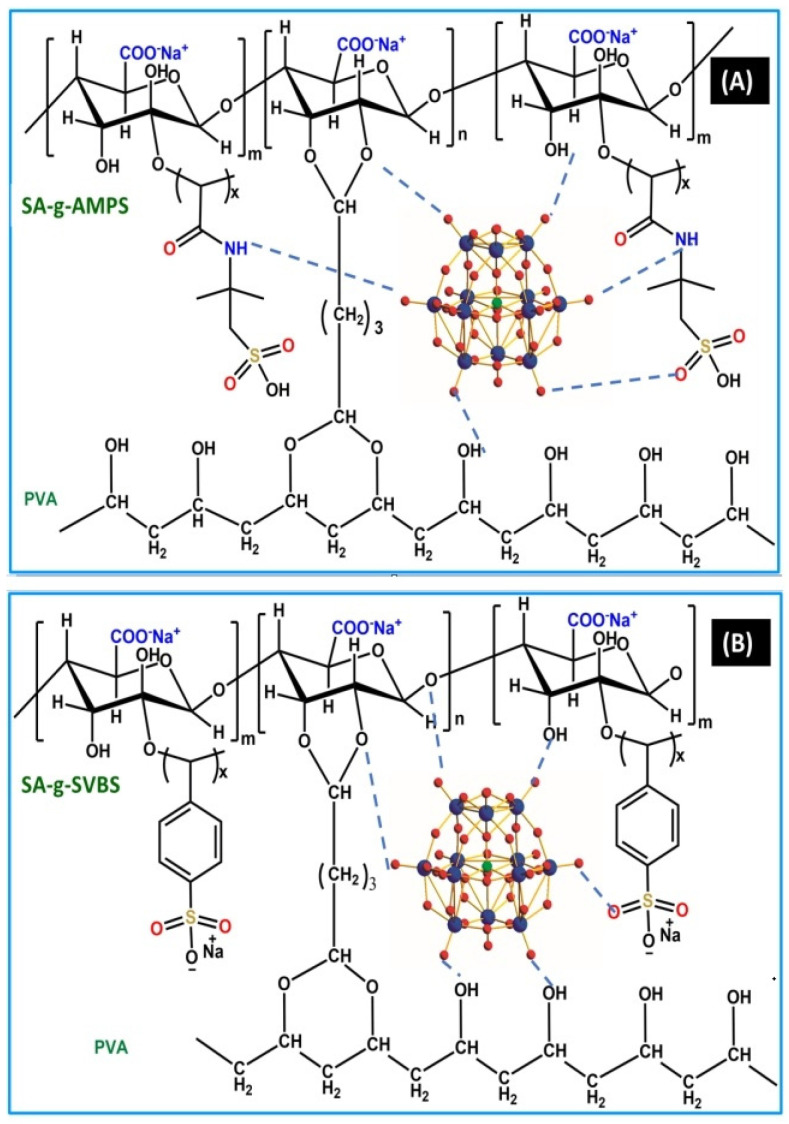
Schematic representation of PMA embedded in PSAAM: (**A**) PSASB (**B**) PEMs.

All membranes are made from blends of sulfonated SA (SA-g-AMPS/SA-g-SVBS) and PVA. Two grams of SA-g-AMPS/SA-g-SVBS was dissolved in 40 mL of distilled water in a separate beaker. Fixed concentrations of PVA (3 g) aqueous solution were made at 80 °C, and the resulting solution was cooled to room temperature after full dissolution of PVA. The two polymer solutions were mixed and stirred for 12 h. The resulting blend solution was allowed in rest mode for 2 h in order to eliminate any bubbles formed; finally, this solution was then casted on a clean glass plate so that the solvent can evaporate. The dried membranes were crosslinked with an acetone:water (50:50) mixture bath comprising 2.5 mL GA as the cross-linker and 2.5 mL HCl as the catalyst. These membranes are known as pristine PEMs, namely PSAAM and PSASB. Additionally, composite PEMs are made by adding 10% PMA to polymer mix solutions prior to casting, and these membranes are known as PSAAM-PMA and PSASB-PMA (Scheme 1).

### 2.3. Swelling Studies 

Equilibrium water uptake capacities of PEMs were performed in DD water at 30 °C for 12 h. Dried PEM (*W_d_*) of 0.10 g was immersed into 50 mL of DD water and removed after 12 h, and the surface of PEM wiped with tissue paper and weighed (*W_e_*). The percentage of equilibrium swelling ratio (%*S_e_*) was calculated by the following equation.

(1) $$\%S_{e}=\frac{W_{e}-W_{d}}{W_{d}}\times 100$$

### 2.4. 5-Fluorouracil (5FU) Encapsulation and Release Studies 

5*FU* entrapment was performed using the equilibrium swelling method [13] by allowing PEMs to swell 12 h in alkaline 5*FU* solution, and then the 5*FU* entrapped PEMs were dried. These dried membranes were crushed in agate mortar by using 5 mL of DD water to extract trapped 5*FUs*. Then, 5*FU* was estimated by using a UV-vis spectrophotometer (LABINDIA, UV-3092) at λ_max_ 270 nm. The 5*FU* encapsulation efficiency (%5*FU* EE) was calculated by using the equations below.

(2) $$\%5FU\;entrapment=\left(\frac{Amount\;of\;5FU\;entrped\;in\;PEM}{Amount\;of\;PEM\;taken}\right)\times 100$$

(3) $$\%5FU\;EE=\left(\frac{Actual\;entrapemnt\;of\;5FU}{Theoritical\;entrapment\;of\;5FU}\right)\times 100$$

The release of 5*FU* from drug trapped PEMs was studied by using a dissolution tester apparatus (LABINDIA, DS-8000) at 37 °C in pH 1.2 and 7.4 buffer media with 100 rpm. Drug release was analyzed at appropriate intervals and estimated with UV-Vis spectrophotometer. The resulting data were fitted with drug release kinetics models [46], i.e., Korsmeyer–Peppas, zero order, first order, Higuchi and Hixson–Crowell models.

(4) $$\frac{M_{t}}{M_{\infty }}=Kt^{n}$$

(5) $$Q=Q_{o}-K_{o}t$$

(6) $$lnQ=lnQ_{o}-K_{1}t$$

(7) $$M_{t}=K_{H}^{\frac{1}{2}}t$$

(8) $$Q_{t}^{\frac{1}{3}}=Q_{o}^{\frac{1}{3}}-K_{c}t$$

### 2.5. Sorption Studies of Cu(II) Ions 

The Cu(II) ion stock solution (1 g/L) was prepared by dissolving 3.8017 g of Cu(NO_3_)_2_.3H_2_O in 100 mL DD water. The required concentrations (400–1100 ppm) of metal ions were prepared by dilution of stock solution with DD water, and pH was adjusted to 5.5 with 0.01 M NaOH and HCl. Sorpton studies of Cu(II) ions were conducted by batch experiment [30,31]. Briefly, 40 mg of membrane was weighed and placed in Cu(II) ion aqueous solution; the samples were shaken at 30 °C with 200 rpm for 4 h. The concentrations of Cu(II) ion solutions were measured with atomic absorption spectroscopy (SHIMADZU, AA-6880). The results are computed with the following expression:

(9) $$Q_{e}=\left(C_{o}-C_{e}\right)\frac{V}{M}$$

where *C_o_* and *C_e_* are the initial and residual concentrations of Cu(II) ion in liquid phase, respectively. *V* is the volume of the Cu(II) ion solution (L), and *M* is the amount of PEM (g).

### 2.6. Ion Exchange Capacity, Oxidative Stability, Proton Conductivity and Methanol Permeability Studies 

The ion exchange capacity (IEC) of PEMs was determined by simple acid-base titration method. Briefly, the known amount of the PEM was soaked in 50 mL of aqueous NaCl solution (3 M) for 24 h. Subsequently, 10 mL of solution was titrated against the NaOH solution (0.01 N). IEC was calculated by using the following expression.

(10) $$IEC=\frac{Volume\;of\;NaOH\;consumed-Concentration\;of\;NaOH}{Dry\;weight\;of\;the\;PEM}$$

PEMs were analyzed for oxidative stability in 20 mL of Fenton’s reagent at 30 °C for 6 h. The percentages of weight loss of PEMs were calculated by measuring before and after the reagent treatment.

The proton conductivity of PEMs was determined by the four-probe impedance technique using the electrochemical impedance analyzer (Biologic SP-200) [47]. Fully hydrated PEMs were sandwiched into Teflon blocks, which are equipped with Pt probes. Impedance was measured at 30 °C with 98% humidity. The conductivity of PEMs was calculated from PEM resistance (*R*) by using the following equation:

(11) $$\sigma =\frac{L}{RA}$$

where *σ* is the conductivity of PEM in S/cm, *L* is the thickness of the PEM and *A* is the cross sessional area of the PEM in cm^2^.

Methanol permeability of PEMs was performed [48] by solution-diffusion mechanisms using a Teflon diaphragm diffusion cell, where two reservoirs (approximately 50 mL each) are separated by vertical membrane (effective area 3.14 cm). Permeate methanol concentration was measured by a refractometer AR4 (A. Kruss Optronic, Germany) and calculated using the below equation:

(12) $$P=\frac{C_{b}V_{b}X}{C_{a}AT}$$

where *C_a_*, *C_b_* and *V_b_* are concentrations of methanol in the receptor compartment, concentration of methanol in the donor compartment and receptor volume, respectively. *A* is the effective area of the membrane, and *T* is the time consumed when equilibrating the system.

### 2.7. Characterization

^1^H Nuclear magnetic resonance spectroscopy (Brucker A vance 500 MHz) was used to analyze the chemical structure of sulfonate graft SA copolymers (SA-g-AMPS and SA-g-SVBS). Fourier transform infrared studies, FTIR (Perkin Elmer, Spectrum Two) and attenuated total reflection-FTIR analysis (Bruker, Alpha-II, Eco-ATR) were used to characterize the graft copolymers and PEMs’ chemical structure, encapsulation of 5FU in PEMs and sorption of Cu(II) ion by the PEMs. Scanning electron microscopy and energy dispersive X-ray study (JOEL, JSM IT500) were used to characterize the surface morphology of PEMs and elemental composition of the surface of the PEMs, respectively. Differential scanning calorimetry study of graft copolymer was performed with TA instruments (STA, Q600). X-ray diffraction studies (Rigaku, Miniflex 600) of PEMs and PMA incorporated with PEMs were characterized to analyze PMA dispersity in the polymer network.

## 3. Results and Discussion

### 3.1. Synthesis of Sulfonate Grafted Sodium Alginates

Sulfonate grafted sodium alginates were synthesized from sodium alginate (SA) with 2-acrylamide-2-methyl-1-propanesulphonic acid (AMPS) and sodium 4-vinylbenzene sulfonate (SVBS) by simple free radical polymerization using ceric ammonium nitrate (CAN) and ammonium persulphate (APS), respectively (Scheme S1). SA-g-AMPS and SA-g-SVBS were characterized by ^1^H NMR, FTIR, XRD and DSC studies (Figures S1–S4). The plausible chemical structure of SA-g-AMPS and SA-g-SVBS is shown in Scheme S2. Graft copolymeric reaction parameters are optimized with respect to the concentration of monomer, initiator, temperature and time; the results are presented in (Tables S1 and S2; Figures S5 and S6). The maximum grating and grafting efficiency of SA-g-AMPS are 62.06% and 42.78%, respectively, at optimized reaction conditions, i.e., reaction temperature 60 °C, AMPS concentration 3.5 mmol, CAN concentration 0.11 mmol and reaction time 120 min. The maximum grafting and grafting efficiency of SA-g-AMPS are 84.38% and 65.44%, respectively, at optimized reaction conditions, i.e., reaction temperature 60 °C, SVBS concentration 3.5 mmol, APS concentration 0.036 mmol and reaction time 120 min.

### 3.2. FTIR Studies

FTIR spectra of SA, SA-g-AMPS and SA-g-SVBS are presented in the Figure S2. Pure SA has exhibited significant peaks at 3435, 2924, 1614 and 1095 cm^−1^ for stretching vibrations of -OH, -CH, -C=O and -CH-O-CH- functional groups. Other peaks appeared at 1414 and 1304 cm^−1^ for scissoring and bending vibrations of -OH. In addition to SA peaks with strong intensities, FTIR spectra of SA-g-AMPS exhibited significant peaks at 1665, 1409 and 1218 cm^−1^ for -NH and S=O groups, respectively. Moreover, FTIR spectra of SA-g-SVBS exhibited significant peaks at 2924 and 2938 and 1409 and 1218 cm^−1^ for -C=C (aromatic) and S=O groups, respectively.

**Figure 1:**
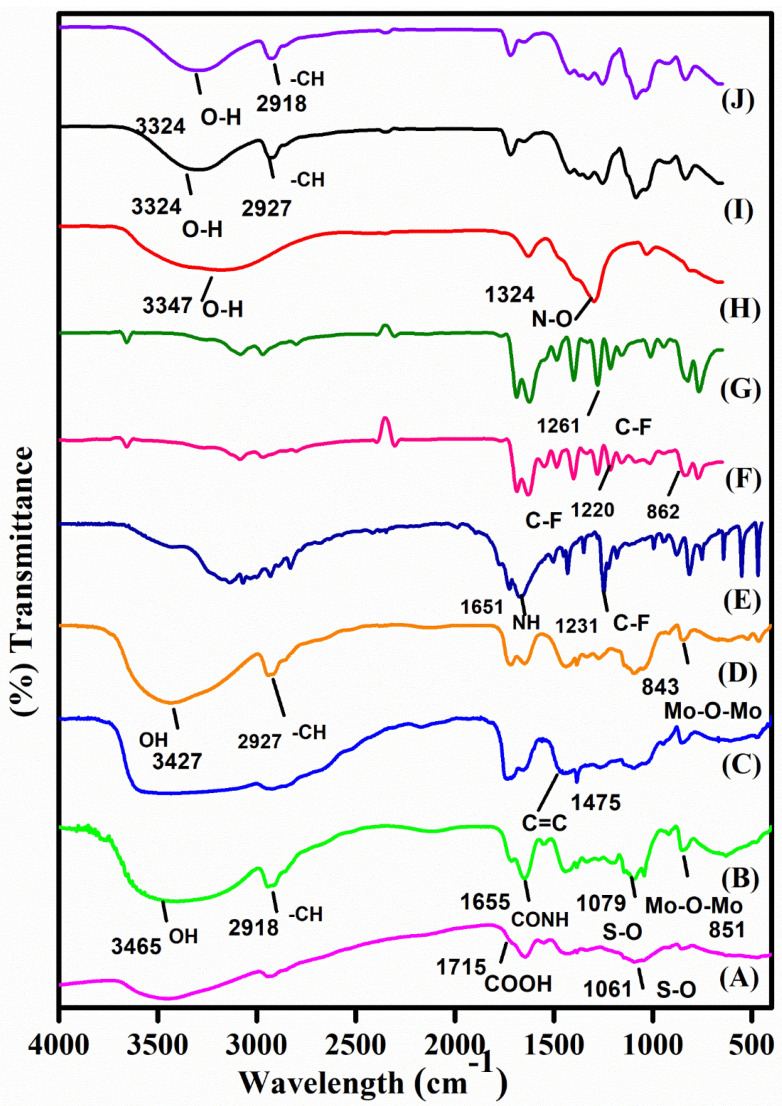
FTIR spectra of PSAAM (**A**), PSAAM-PMA (**B**), PSASB (**C**), PSASB-PMA (**D**) and 5FU (**E**); ATR-FTIR spectra of PSAAM-5FU (**F**), PSASB-5FU (**G**), Cu(NO_3_)_2_.3H_2_O (**H**) PSAAM-Cu(II) (**I**) and PSASB-Cu(II) (**J**).

FTIR and ATR-FTIR spectra of PSAAM (A), PSAAM-PMA (B), PSASB (C), PSASB-PMA (D), 5FU (E), PSAAM-5FU (F), PSASB-5FU (G), Cu(NO_3_).3H_2_O (H) PSAAM-Cu (II) (I) and PSASB-Cu(II)(J) are shown in the Figure 1. From Figure 1A,C the responsible functinal groups of -OH (streching) of PVA and SA, -COO^−^ of SA, -CO-NH of AMPS, C=C of SVBS and -S=O of AMPS and SVBS are observed at 3465, 1715, 1655, 1475 and 1061/1079/1040 cm^−1^, respectively. Moreover, -C-H (bending) was observed at 1420 cm^−1^, and C-C (symmetric) was observed at 2922 cm^−1^ in the PEM network. In addition, the crosslinking reaction between the aldehydic group of glutaraldehyde and hydroxyl groups of PVA/SA evidenced for C-O-C at 1090 cm^−1^. In the case of PMA incorporated PEMs (Figure 1B,D) the peaks at 851 and 843, 966, 783 and 1060 cm^−1^ are attributed to M=O_d_, M-O_b_-M, M-O_c_-M and O=P=O, respectively [41,43]. From Figure 1E, the significant vibrational peaks observed at 1665, 3140, 3067 and 1262/861 cm^−1^ functional groups of 5FU are C=O, N-H, =C-H and C-F. However, in the case of 5FU encapsulated PEMs, they exhibited peaks at 1230 cm^−1^ for C-F, while the other functional groups of 5FU are overlapped with the PEM network. Compared to the spectra of pristine PEMs before and after adsorption of the Cu(II) ion, the adsorption peaks at 3465,1715, 1655, 1475 and 1061/1079/1040 cm^−1^ corresponding to -OH stretching, C=O stretching, C-N stretching, C=C stretching and S=O stretching either decreased in intensity or shifted the peaks from their respective adsorption. Hence, the presence of nitrogen, oxygen and sulphur atoms in the amide, carboxyl/hydroxyl and sulfone groups are responsible for the sorption of Cu(II) ions attachment [30,31].

### 3.3. XRD Studies

**Figure 2:**
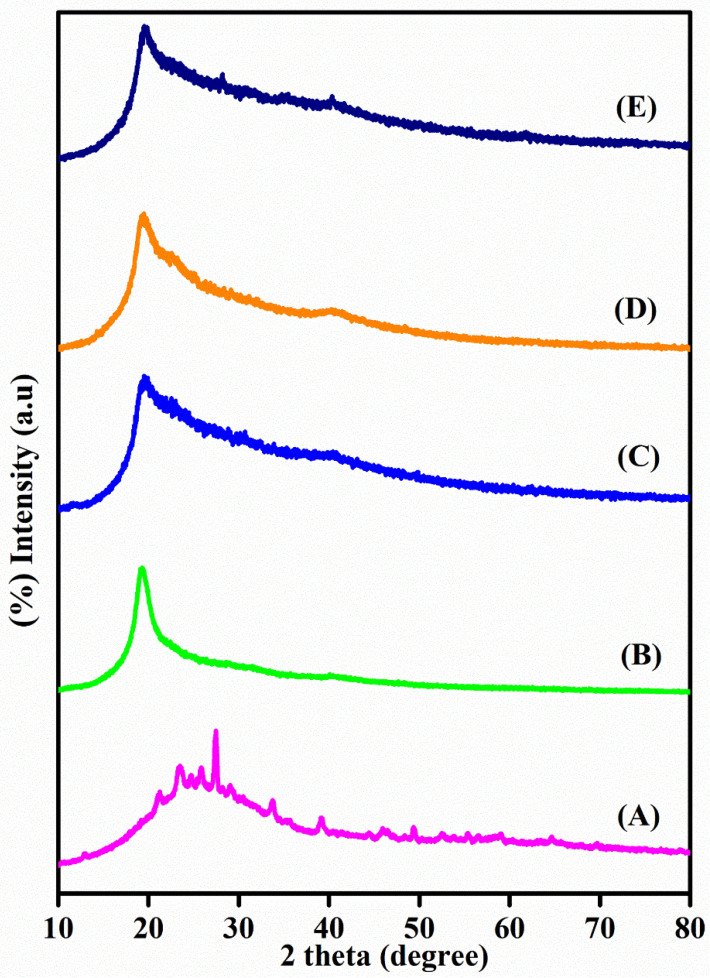
XRD patterns of phosphomolybdic acid (**A**) PSAAM (**B**), PSASB (**C**), PSAAM-PMA (**D**) and PSASB-PMA (**E**).

XRD patterns of phosphomolybdic acid (A) PSAAM (B), PSASB (C), PSAAM-PMA (D) and PSASB-PMA (E) are shown in the Figure 2; XRD results are pesented in the range of 10° to 80° of 2θ values. Prestine PEMs have exhibited semi crysttaline peaks at 2θ of 20° [49]. From Figure 2A, PMA exhibitted characteristic crystalline peaks at 22, 27, 33 and 37 for (210), (221), (311) and (321), respectively [50]. However, PMA incorporated PEMs are not shown such crysttaline peaks, which indicates that PMA is molecularly dispersed in PEMs.

### 3.4. SEM and EDAX Studies

**Figure 3:**
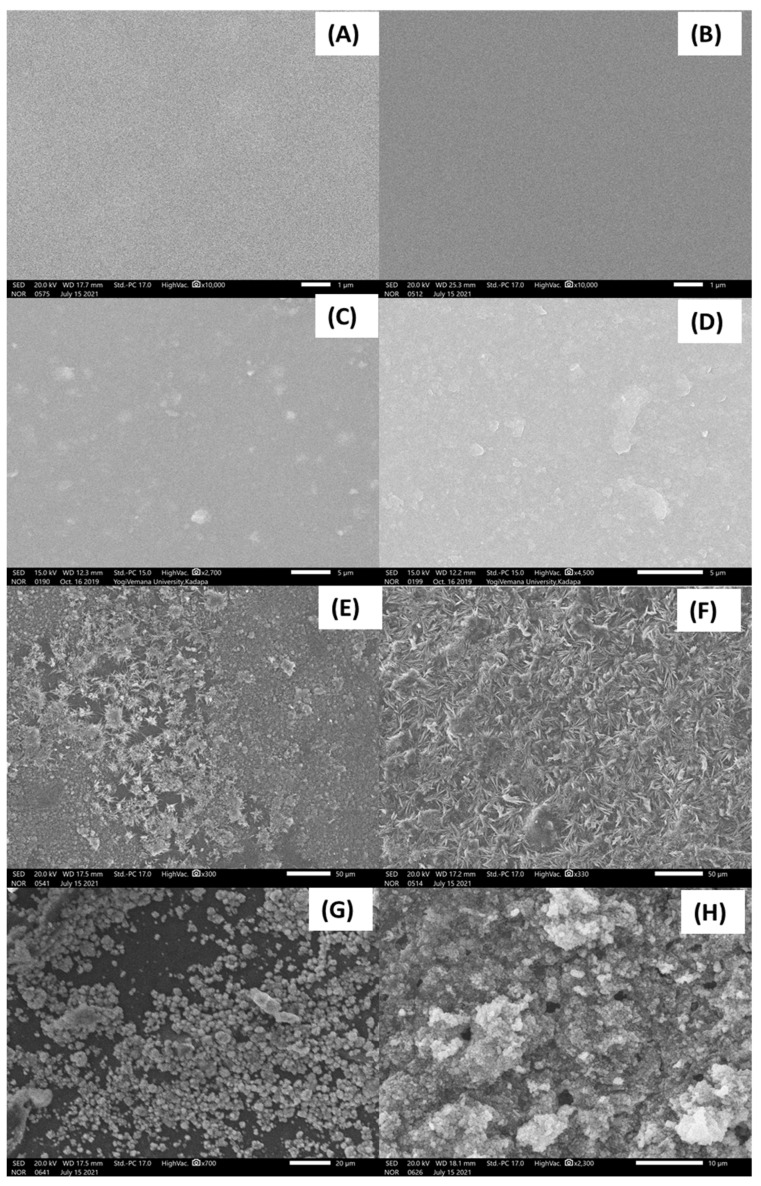
SEM images of PSAAM (**A**), PSASB (**B**), PSAAM-PMA (**C**), PSASB-PMA (**D**), PSAAM-5FU (**E**), PSASB-5FU (**F**), PSAAM-Cu (II) (**G**) and PSASB-Cu(II) (**H**).

SEM images of pristine PEMs, PMA incorporated PEMs, 5FU encapsulated PEMs and Cu(II) sorbed PEMs are presented in Figure 3. PSAAM and PSASB membranes displayed smooth surfaces with non porous structure. However, PMA incorporated PSAAM and PSASB membranes showed rough surfaces and homogeneous distribution. In general, aqueous Cu(II) ions are sorbed by PEMs due to the interactions between the functional groups (-SO_3_^−^, -CONH, -OH and -COO^−^) and sorbed Cu(II) ions. Figure 3E,F show the images of 5FU sorbed PEMs; the surface is very rough, and this may be because of the recrystalisation of encapsulated 5FU during the drying process of PEM. Figure 3G,H shows the images of Cu(II) ion sorbed PEMs, and many well-dispersed Cu(II) ion salts formed on the sufrace of the PEM, which resulted in the rougher surface.

**Figure 4:**
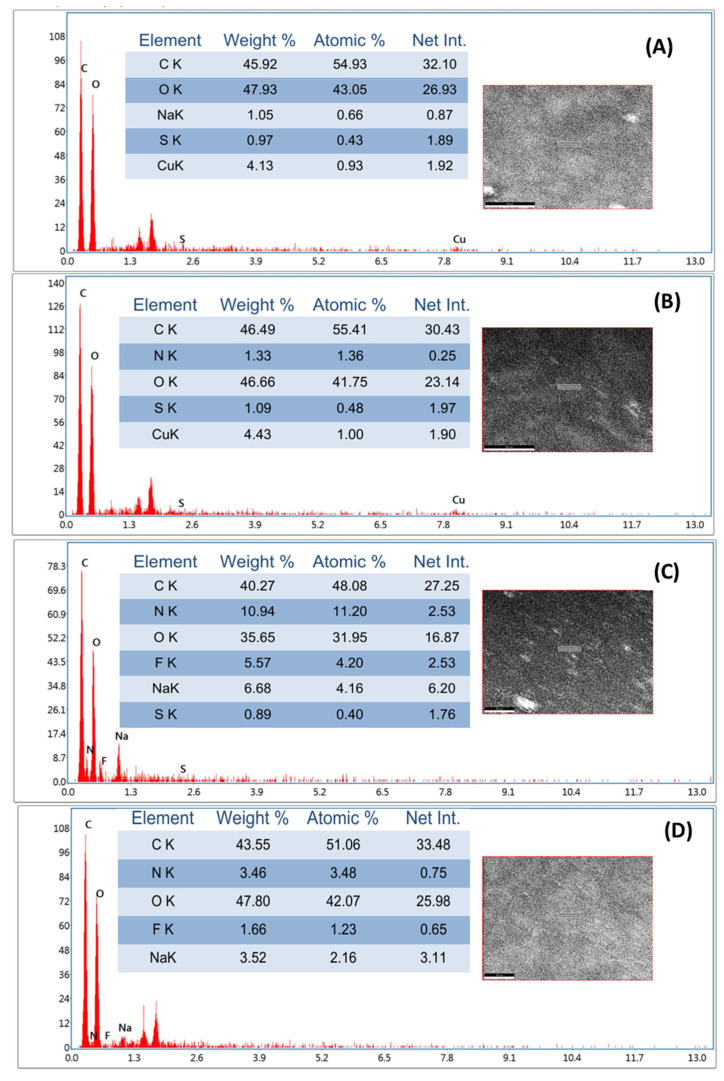
EDAX patterns of PSAAM-5FU (**A**), PSASB-5FU (**B**), PSAAM-Cu(II) (**C**) and PSASB-Cu(II) (**D**).

Figure 4 showing the EDAX spectra analysis of 5FU encapsulated PEMs and Cu(II) ion adsorbed PEMs confirmed that 5FU and Cu(II) ions are distributed inside as well as on the surface of the polymer network consisting of O, N and S. EDAX results of PSAAM-5FU and PSASB-5FU demonstrate that PEMs consisted the elements C, O, N, S, Na and F; however, the weight percentages of fluorine was 5.57 and 1.66 based on the intensity of the fluorine peak. Moreover, EDAX results of PSAAM-Cu(II) and PSASB-Cu(II) demonstrate that PEMs consisted of elements C, O, N, S, Na and Cu; however, the weight percentages of copper 4.13 and 4.43 were based on the intensity of the copper peak.

### 3.5. Equilibrium Swelling Studies

Swelling is one of the important characteristics of polymer membrane, which helps us understand the water holding capacity. It depends on the nature of functional groups and the type of dopant present in the matrix. Therefore, swelling studies were performed for both pristine PEMs and PMA incorporated membranes. The %Equilibrium swelling ratios (%S_e_) are presented in Table 1, i.e., 369, 258, 300 and 169 for PSAAM, PSASB, PSAAM-PMA and PSASB-PMA, respectively. The results indicate that %S_e_ decreased with the incorporation of PMA. This may be due to the formation physico-chemical interaction between the polymer and inorganic polyacid [51].

### 3.6. 5-Fluorouracil Drug Delivery

**Figure 5:**
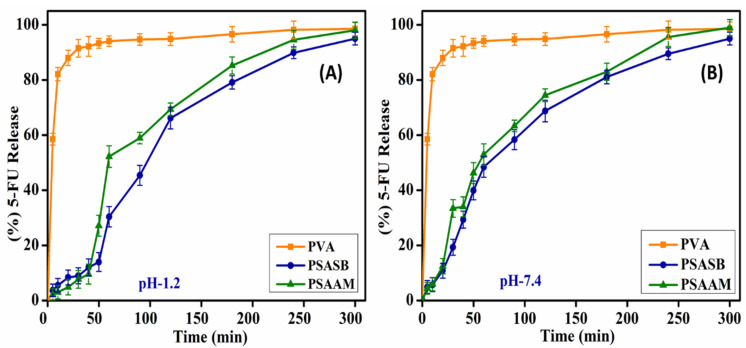
FU release characteristics of PVA, PSASB and PSAAM in pH 1.2 (**A**) and 7.4 (**B**) at 37 °C.

5FU is a potential chemotherapeutic agent and is extensively used for various types of cancers. In the present study, 5FU is physically encapsulated by the equilibrium swelling method; hence, it is essential to understand the release kinetics of 5FU. %Encapsulation efficiencies for PSAAM and PSASB are 78 ± 2.3 and 66 ± 4.7, respectively. The 5FU release profiles of PEMs at pH 1.2 and 7.4 at 37 °C are presented in Figure 5. Table S3 shows the various drug release models (zero order, first order, Higuchi, Hixson–Crowell and Korsmeyer–Peppas) fitted with the 5FU release kinetics data. The best R^2^ observed for all the kinetic models, however, is maximum Hixson–Crowell and Korsmeyer–Peppas models at pH 1.2 with slope value (n) ranges from 0.309 to 0.94, i.e., 0.309 < n < 0.94. These results demonstrate that drug release followed the non-Fickian drug transport mechanism, i.e., the dissolution of water-soluble drug in PEM network [46]. Similar mechanisms are also observed in the case of pH 7.4.

### 3.7. Copper(II) Ion Removal

**Figure 6:**
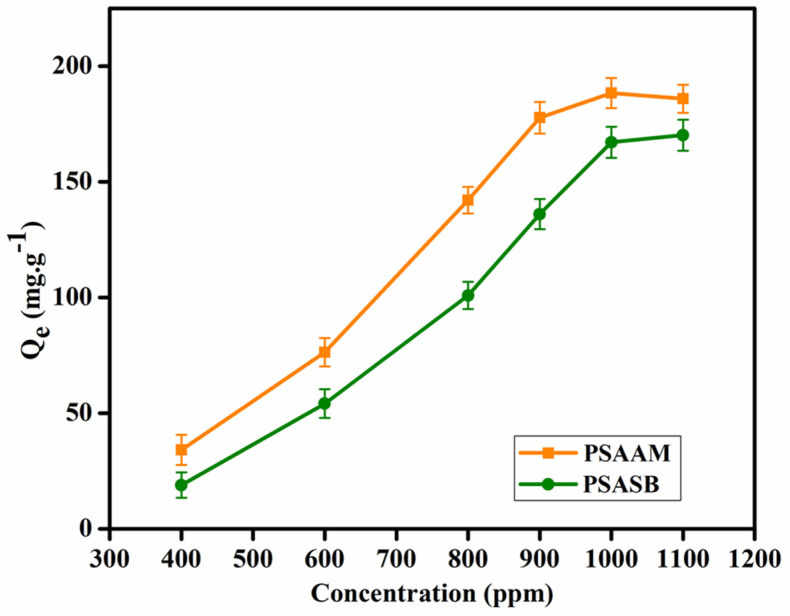
Cu (II) ion adsorption by PSASB and PSAAM in pH 5.5 at 30 °C.

Figure 6 shows the effect of Cu(II) ion concentration on PSAAM and PSASB PEMs in pH = 5.5 aqueous meadia at 30 °C. The presence of functional groups, such as croboxyl group of SA, amide and sulfone group of AMPS/SVBS and hydroxyl group of PVA, is a key factor in either chemisorption or physisorption of metal ions. In the present study, the maximum adsorption capacities were observed in pH 5.5 at 30 °C, and 188.91 and 181.22 mg.g^−1^ were achieved for PSAAM and PSASB, respectively. The present results compared with the literature with respect to adsorbant to adsorbate (mg.g^−1^) are shown in Table 2. Sudha Vani et al. synthesized novel sodium alginate-gelatin blend membranes and studied their toxic metal ion (Cu(II) and Ni(II)) removal capacities [4]. The Q_e_ values are reported to be 43.51 (Cu(II)) and 240.64 (Ni(II)). Moreover, the development of membranes from polyaniline and poly(acrylic acid)-g-sodium alginate/gelatin was also reported [31]. The Q_e_ values of this study were reported as 53.29 for Cu(II) and 129.28 for Ni(II). Zhao et al. developed sodium alginate-polyethylene glycol oxide-nano materials based composite gels for the removal of heavy metal ions such as Cu(II) and Cd^2+^ from polluted water [52]. In this study, the fabricated materials showed the adsorption capacity (Q_m_) for Cu(II) and Cd(II) as 6.78 and 3.43 with the removal rates 96.8% (Cu(II)) and 78% (Cd(II)). Li et al. synthesized calcium alginate immobilized kaolin using the sol-gel process, and it was used for the removal of Cu(II) from its aqueous solutions [53]. The Q_e_ value related to the removal of Cu(II) using alginate immobilized kaolin was reported to be 53.63. Yang et al. established calcium alginate beads as adsorbent materials for the removal of heavy metal ions such as Cu(II), Zn(II), Ni(II) and Cd(II) [54]. The Q_e_ values were reported for Cu(II)as 140.55, Zn(II) as 174.60, Ni(II) as 114.69 and Cd(II) as 216.82. Lai et al. synthesized cellulose (derived from orange peel (OPC) and banana peel (BPC)) immobilized calcium alginate (CA) materials for the removal of Cu(II), Pb(II) and Zn(II) ions from aqueous solutions [55]. The Q_e_ values of CA-OPC were reported as 166.67 (Cu(II)), 128.20 (Pb(II)) and 156.25 (Zn(II)). The Q_e_ values of CA-BPC were reported as 163.93 (Cu(II)), 121.95 (Pb(II)) and 153.85 (Zn(II)). Petrovič and Simonič studied the fabrication and heavy metal ion removal applications of calcium alginate-immobilized Chlorella sorokiniana materials [56]. These materials are studied for the removal of Cu(II), Ni(II) and Cd(II), and the Q_e_ values are reported as 179.90 (Cu(II)), 86.49 (Ni(II)) and 164.50 (Cd(II)).

### 3.8. Ion Exchange Capacity, Oxidative Stability Proton Conductivity and Methanol Permeability Studies

**Figure 7:**
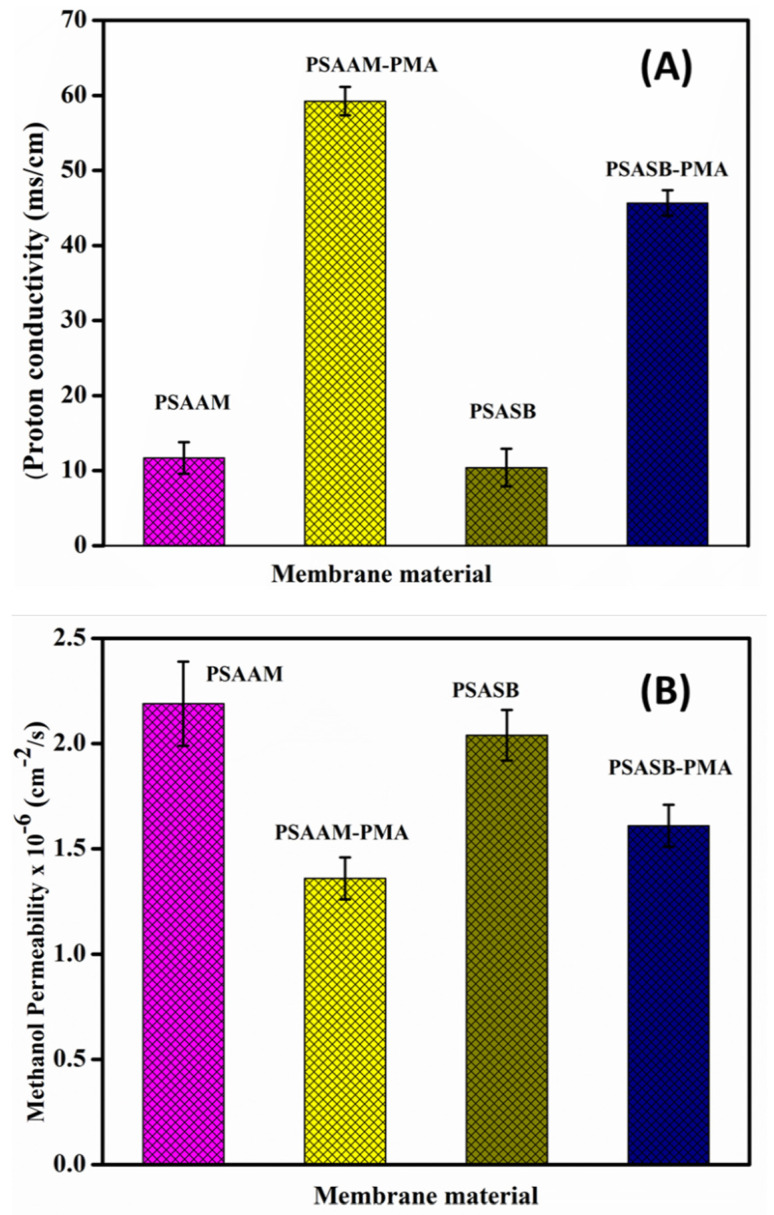
Proton conductivity (**A**) and methanol permeability (**B**) of the PEMs.

In addition to drug delivery and sorption studies of metal ions, fabricated membranes with and without PMA are examined for the suitability to function as PEMs for fuel cells. In this context, %S_e_, ion exchange capacity (IEC), proton conductivity study and methanol permeability of the PEM were tested (Table 1). Figure 7 shows the proton conductivity study and methanol permeability of PSAAM, PSASB, PSAAM-PMA and PSASB-PMA at 30 °C under atmospheric pressure. IEC and %S_e_ are the key factors influencing the proton conductivity study and methanol permeability.

In particular, PEMs are chemically treated with glutaraldehyde and results in the formation of acetal linkage and hydrophobic crosslinks, which protect polymer from dissolving in water and provides stable morphology. The oxidative stability of PEMs was evaluated in Fenton’s reagent at 30 °C, and the results indicate that PMA incorporated PEMs shown significantly high stabilities owing to the dense structure of composite PEMs, which controls the penetration of radicals with polymer chains. This behavior also supports the good mechanical property of the PEMs.

Proton conductivity of PEMs are in the order of 10^−3^ S/cm; however, it significantly increased with incorporation of 10% PMA into the PEMs. This may be due to proton transfer (i.e., in the form of H_3_O^+^, H_5_O_2_^+^ and H_9_O_4_^+^) across the membrane with the help of the functional groups -COO^−^, -CONH and -SO_3_H., where hydronium ion dissociates and forms hydrogen bonds (*Grotthus Mechanism*) [57]. This is also supported by high %S_e_ (over the 100%), i.e., the more hydrophilic nature of PEMs enhances the ionic nature of PEMs [58]. On the contrary, PMA incorporated PEMs (2.19 × 10^−6^ cm^2^/s and 2.04 × 10^−6^ cm^2^/s) demonstrated lower methanol permeability when compared to pristine PEMs (1.36 × 10^−6^ cm^2^/s and 1.61 × 10^−6^ cm^2^/s). This is because of its polyacid *pseudo liquid phase* nature [59,60].

## 4. Conclusions

In summary, sulfonate functionalized sodium alginate-based PEMs were fabricated by the simple solution casting method. Composite PEMs were also fabricated by the incorporation of PMA in the polymer matrix. The fabricated PEMs were used for drug delivery applications of a chemotherapeutic agent (5FU) and adsorption of Cu(II) ions from the aqueous solutions. The drug release kinetics demonstrate that drug release followed the non-Fickian drug transport mechanism. From the adsorption studies, it was found that PEMs possess significant adsorption capacities (188.91 and 181.22 mg.g^−1^ for PSAAM and PSASB, respectively). Fabricated PEMs are well suited for fuel cells as their proton conductive membrane functions as an effective proton transporter with respect to their dual functionalities, i.e., -SO_3_^−^ and -COO^−^. In addition, polyacid (PMA) incorporated PEMs accelerated more proton conductivity. PEMs showed relatively good water management, significant IEC, considerable proton conductivity and low methanol permeability. Therefore, PEMs are eco-friendly, low-cast and promising practical usage materials for delivery, adsorption and transport of molecules or ions for controlled release, precious metal extraction or organic dye removal and fuel cell applications.

## Figures and Tables

**Table 1:** Composition, %S_e_, IEC, proton conductivity and methanol permeability of various PEMs.

Type of Membrane	PVA (g)	SA-g-AMPS/SA-g-SVBS (g)	PMA (g)	%EE of 5FU	Q_e_ mg.g^–1^	%S_e_	IEC	Oxidative Stability (RW%)	Proton Conductivity (mS/cm)	Methanol Permeability (cm^2^/s) (10^−6^)
PSAAM	3	2	0.5	78 ± 2.3	188.91 ± 6.1	369 ± 6.3	0.64 ± 0.09	79 ± 1.5	11.69 ± 2.1	2.19 ± 0.22
PSAAM-PMA	3	2	0.5	--	--	300 ± 4.9	0.69 ± 0.12	93 ± 2.1	59.23 ± 1.9	1.36 ± 0.12
PSASB	3	2	0.5	66 ± 4.7	181.22 ± 6.7	258 ± 7.1	0.57 ± 0.11	82 ± 3.4	10.41 ± 2.5	2.04 ± 0.12
PSASB-PMA	3	2	0.5	--	--	169 ± 4.8	0.84 ± 0.16	94 ± 2.2	45.66 ± 1.7	1.61 ± 0.14

**Table 2:** Comparison of maximum adsorption capacity of Cu(II) ion by SA based materials.

Sl. No.	Name of the Polymer	Q_e_ (mg.g^–1^)	Ref.
1.	Sodium alginate-polyethylene glycol oxide-nano materials	6.78	[51]
2.	Sodium alginate-gelatin	43.51	[4]
3.	Polyaniline and poly(acrylic acid)-g-sodium alginate/gelatin	53.29	[31]
4.	Calcium alginate immobilized kaolin	53.63	[52]
5.	Sodium alginate	140.55	[53]
6.	Calcium alginate (CA) immobilized range peel cellulose (OPC), banana peel cellulose (BPC)	CA-OPC: 166.67 CA-BPC: 163.93	[54]
7.	Calcium alginate-immobilized Chlorella Sorokiniana	179.90	[55]
8.	PSASB	181.22	Present work
9.	PSAAM	188.91	Present work

## Data Availability

Not applicable.

## Supplementary Materials

The following are available online at https://www.mdpi.com/article/10.3390/polym13193293/s1, ^1^H NMR, FTIR, XRD and DSC studies of graft copolymers (Figures S1–S4); graft copolymerization flow chat and reaction mechanism (Schemes S1 and S2); graft copolymeric reaction parameters (Tables S1 and S2; Figures S5 and S6); and digital photograph of fabricated PEMs (Figure S7).
