# Supplementary material for: Fabrication of Eco-Friendly Polyelectrolyte Membranes Based on Sulfonate Grafted Sodium Alginate for Drug Delivery, Toxic Metal Ion Removal and Fuel Cell Applications
Source: Polymers (Basel). 2021 Sep 27;13(19):3293. doi: 10.3390/polym13193293 (PMC8512661; doi:10.3390/polym13193293)
Supplement: Supplementary file 1 [file polymers-13-03293-s001.zip › polymers-1393886-supplementary.pdf]

Fabrication of Eco-friendly Polyelectrolyte Membranes for Drug Delivery, Toxic Metal Ion Removal and Fuel Cell Applications

R. Vijitha[1], K. Nagaraja[1], Marlia M. Hanafiah[2,3], K. Madhusudana Rao[4], Katta Venkateswarlu[5], Sivarama Krishna Lakkaboyana[6], K.S.V. Krishna Rao[1]*

[1]Polymer Biomaterial Design and Synthesis Laboratory, Department of Chemistry, Yogi Vemana University, Vemana Puram, Kadapa, Andhra Pradesh, India-516005; vijitha.ragaala@gmail.com (V.R.); nagarajakasula33@gmail.com (K.N.)
[2]Department of Earth Sciences and Environment, Faculty of Science and Technology, Universiti Kebangsaan Malaysia, Bangi 43600, Selangor, Malaysia; mhmarlia@ukm.edu.my
[3]Centre for Tropical Climate Change System, Institute of Climate Change, Universiti Kebangsaan Malaysia, Bangi 43600, Selangor, Malaysia
[4]School of Chemical Engineering, Yeungnam University, 280 Daehak-Ro, Gyeongsan, Gyeongbuk, 38541, South of Korea; msraochem@yu.ac.kr
[5]Laboratory for Synthetic & Natural Products Chemistry, Department of Chemistry, Yogi Vemana University, Vemana Puram, Kadapa, Andhra Pradesh, India-516005; kvenkat@yogivemanauniversity.ac.in
[6]Department of Chemical Technology, Chulalongkorn University, Pathumwan, Bangkok 10330, Thailand; svurams@gmail.com

Correspondence: ksvkr@yogivemanauniversity.ac.in; drksvkrishna@yvu.edu.in,
Tel: +91 9704278890

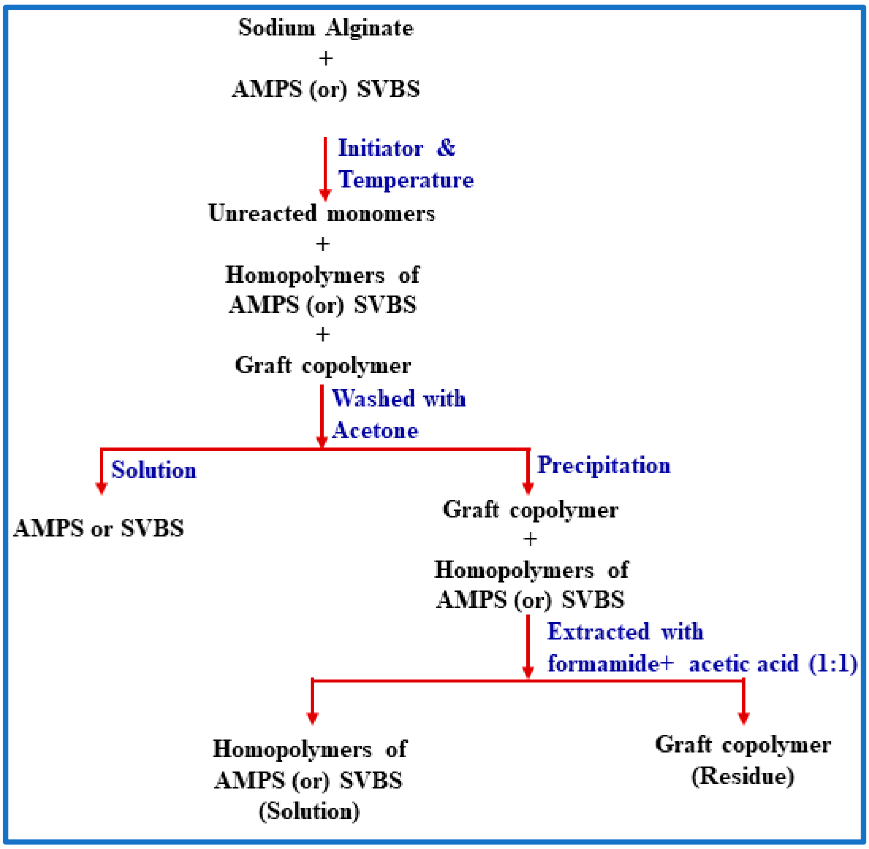

Scheme S1: Flow chart of sulfonate graft SA copolymers preparation and purification.

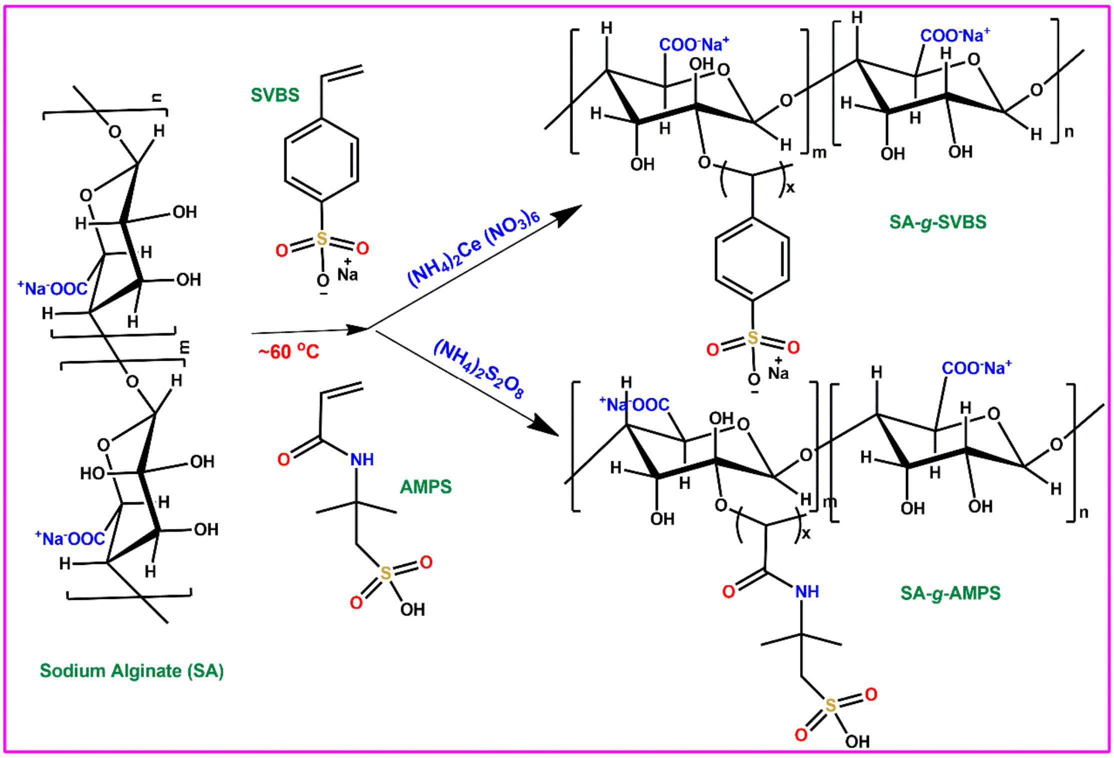

Scheme S2: Plausible schemactic chemistry of graft copolymerisation of AMPS and SBVS on to SA.

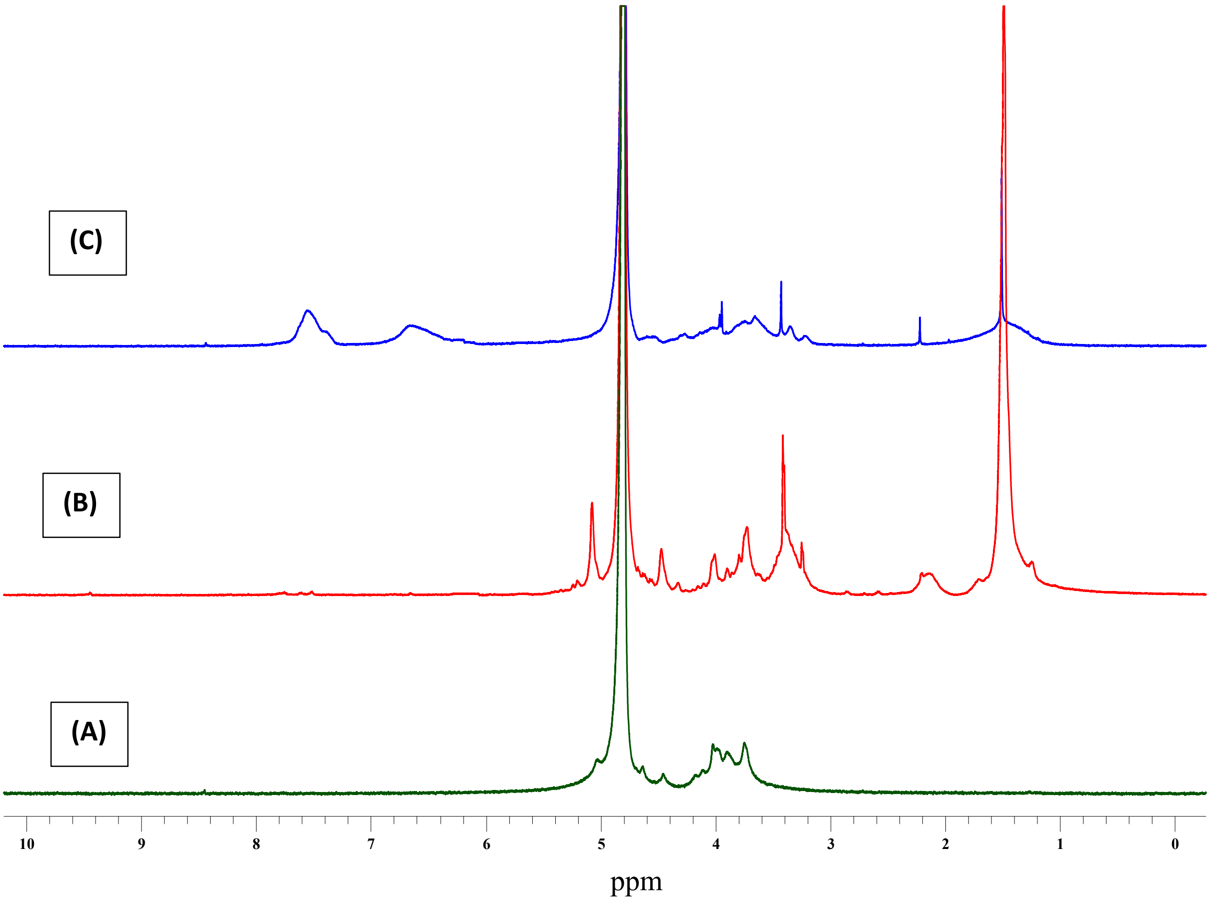

Figure S 1: NMR spectra of pure SA (A), SA-g-AMPS (B) and SA-g-SVBS (C).

NMR Studies.

The ^{1}H NMR spectrum of SA (in D_2O) shows peaks at δ = 5.28–4.57 ppm, 4.45 ppm and 4.24–3.50 ppm. The presence of additional peaks at δ = 3.53–3.13 ppm, 2.29–1.96 ppm and 1.80–1.14 ppm along with the SA backbone peaks at δ = 5.15–3.58 ppm in the ^{1}H NMR spectrum of SA-g-AMPS (in D_2O) copolymer represents the AMPS incorporation on SA. Further, the formation of SA-g-SVBS has been confirmed by the presence of aromatic protons in its ^{1}H NMR spectrum (D_2O) at δ = 7.72–7.27 ppm and 6.89–6.26 ppm along with the SAbackbone peaks at δ = 5.10–3.13 ppm.

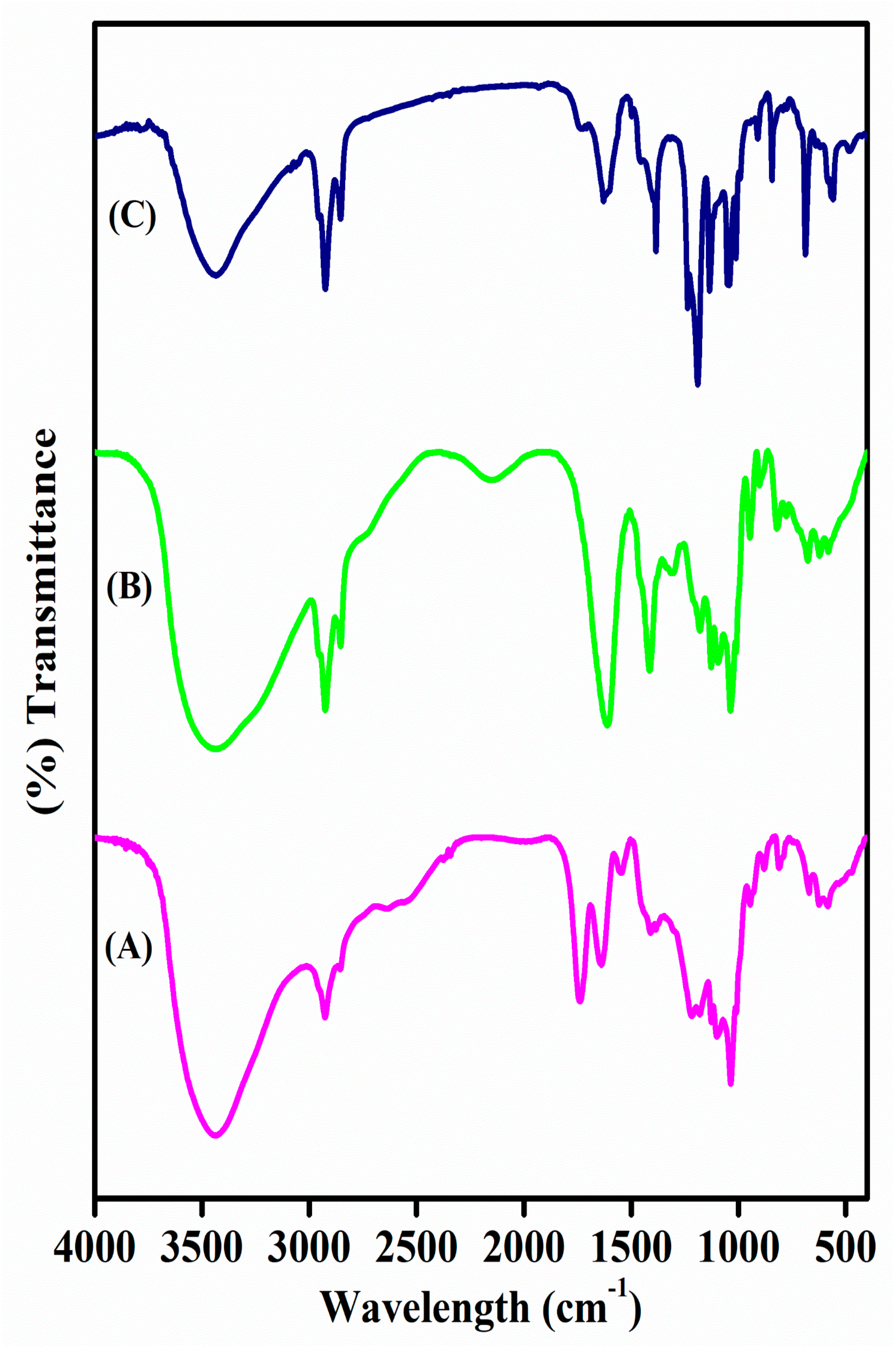

Figure S2: FTIR spectrum of pure SA (A), SA-g-AMPS (B) and SA-g-SVBS (C).

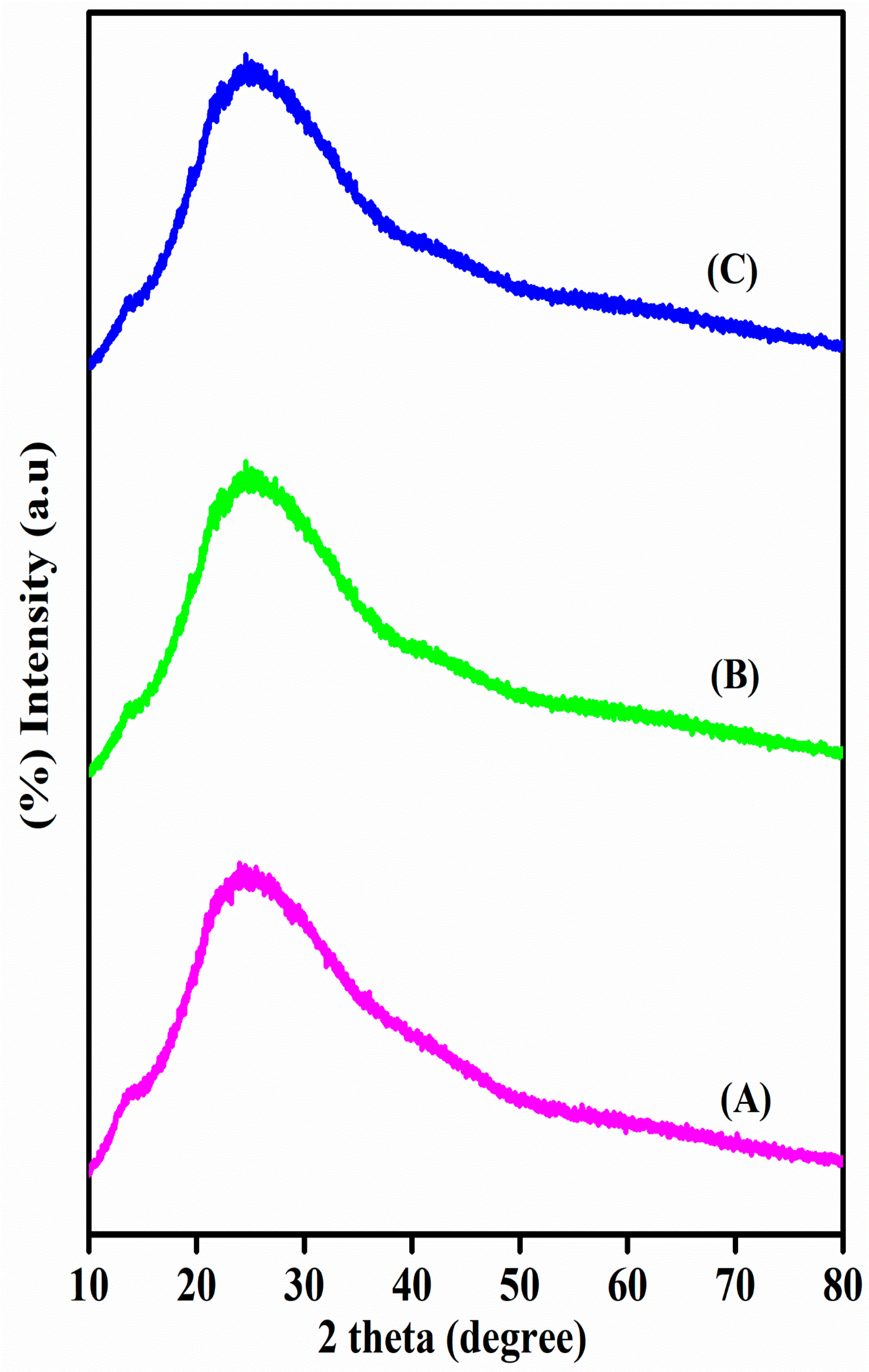

Figure S3: XRD patterns of pure SA (A), SA-g-AMPS (B) and SA-g-SVBS (C).

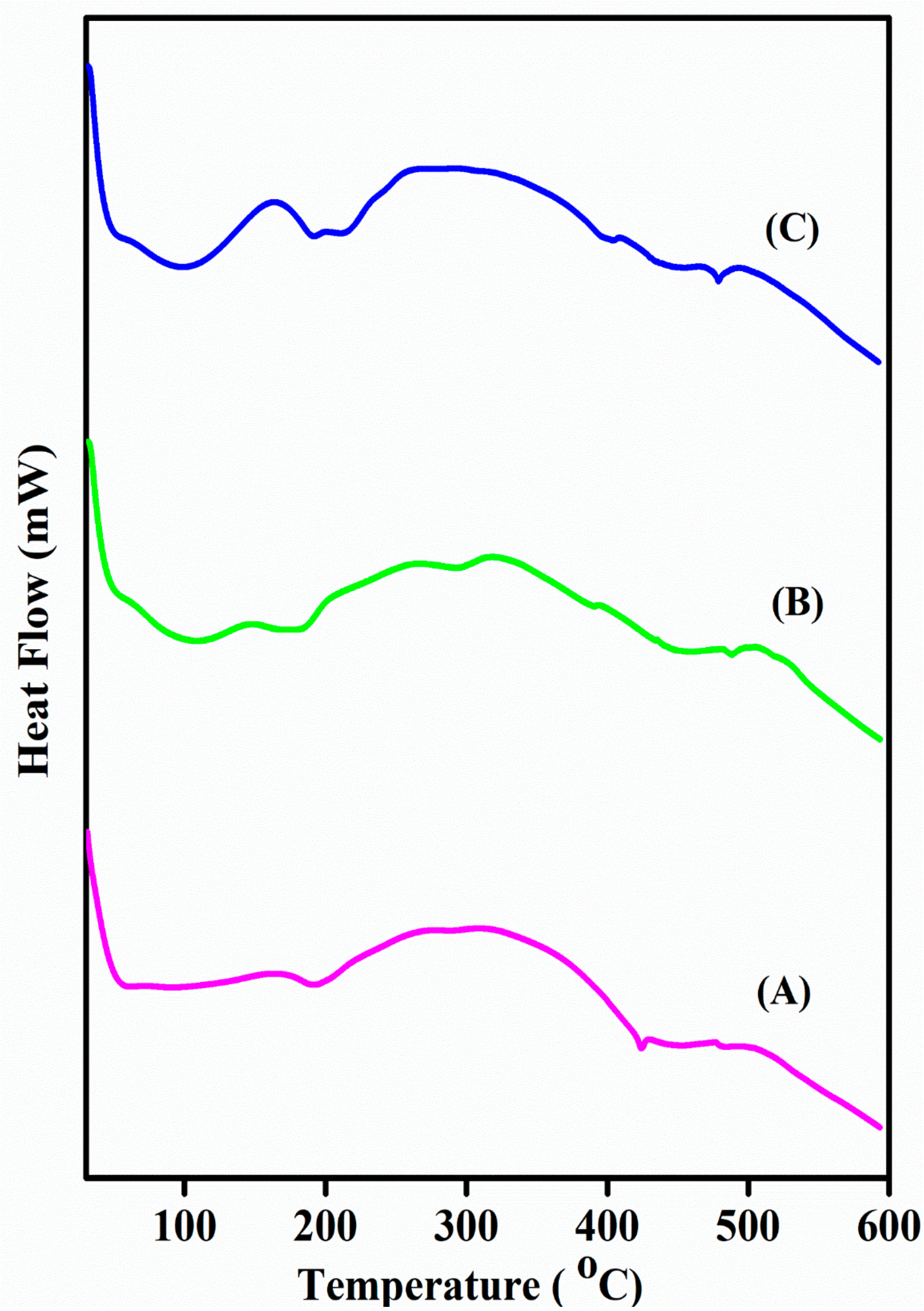

Figure S4: DSC of pure SA (A), SA-g-AMPS (B) and SA-g-SVBS (C).

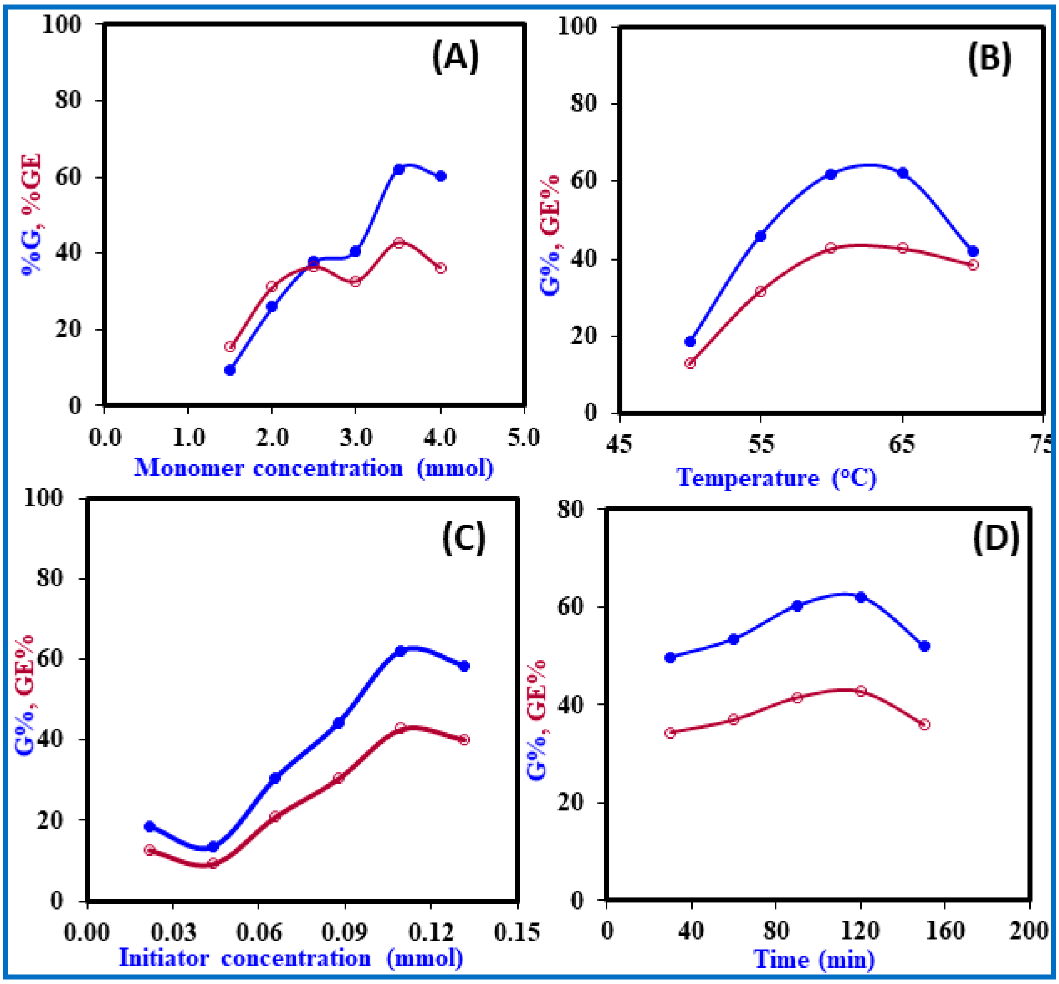

Figure S5: Effect of various reaction conditions on the grafting of AMPS onto the SA, prepared by APS as initiator. **(A)** Effect of monomer concentration [Other reaction conditions: 0.5 g of SA temperature: 60 °C; time: 120 min; initiator concentration 0.09mol.]. **(B)** Effect of reaction temperature [Other reaction conditions: 0.5 g of SA; Monomer concentration: 3.5 mmol; time: 120 min; initiator concentration 0.09 mol.] **(C)** Effect of initiator concentration [Other reaction conditions: 0.5 g of SA; Monomer concentration: 3.5 mmol; time: 120 min; temperature: 60 °C.] **(D)** Effect of reaction time. [Other reaction conditions: 0.5 g of SA; Monomer concentration: 3.5 mmol; initiator concentration 0.09 mol; temperature: 60 °C]

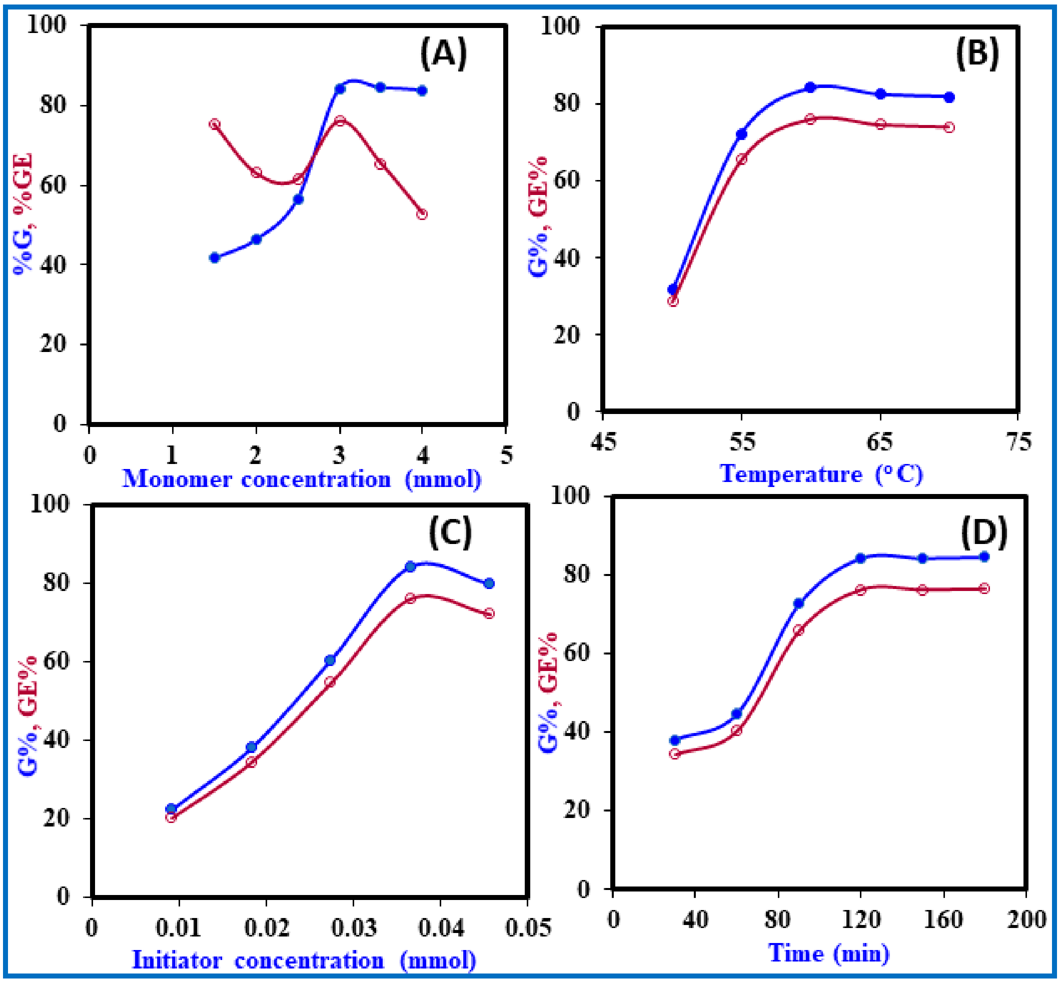

Figure S6: Effect of various reaction conditions on the grafting of SVBS onto the SA, prepared by CAN as initiator. **(A)** Effect of monomer (SVBS) concentration [Other reaction conditions: 0.5 g of SA; temperature: 60 °C; time: 120 min; initiator concentration 0.036 mmol.]. **(B)** Effect of reaction temperature [Other reaction conditions: 0.5 g of SA; Monomer concentration: 3.5 mmol; time: 120 min; initiator concentration 0.036 mmol.] **(C)** Effect of initiator concentration [Other reaction conditions: 0.5 g of SA; Monomer concentration: 3.5 mmol; time: 120 min; temperature: 60 °C.] **(D)** Effect of reaction time. [Other reaction conditions: 0.5 g of SA; Monomer concentration: 3.5 mmol; initiator concentration 0.036 mmol; temperature: 60 °C]

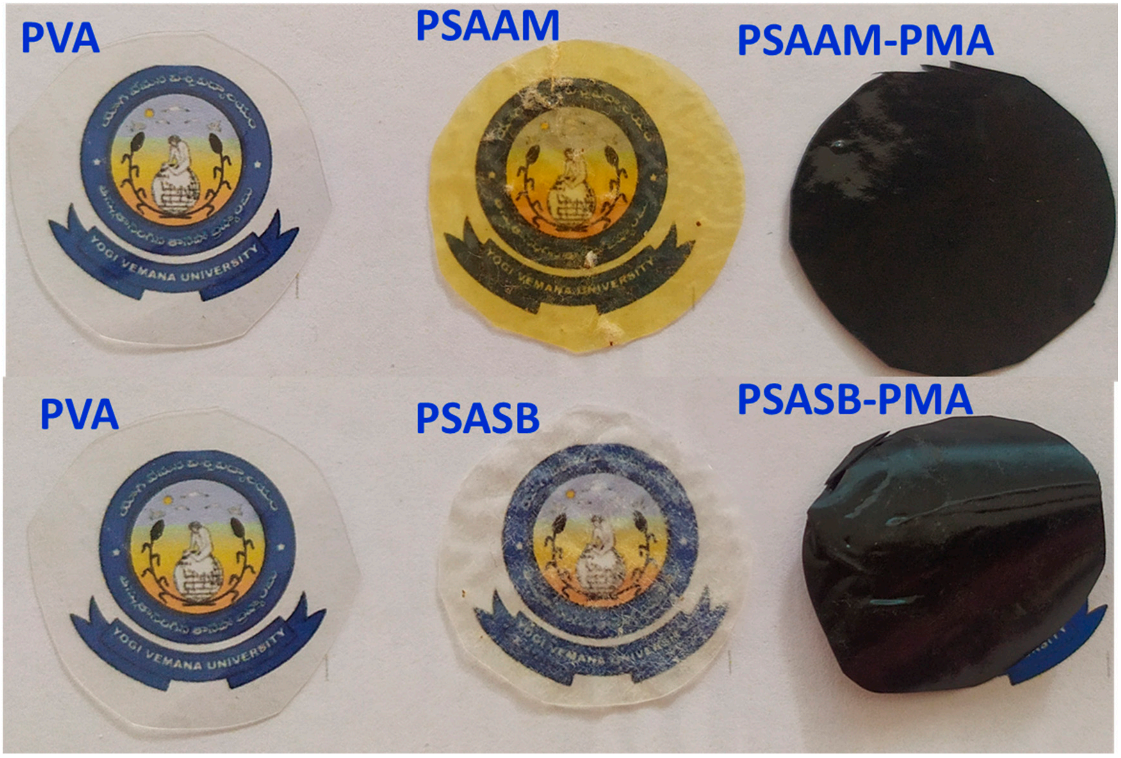

Figure S7: Digital photographs of various Sulfonated SA based PEMs.

Table S1: %Grafting, %grafting efficiency, %conversion, and %yield of PC-g-AMPS graft copolymer prepared by APS as initiator based on effect of various reaction conditions.

	**%Grafting**	**%Grafting Efficiency**	**%Conversion**	**%Yield**
**Temperature (^{o}C)**	**Effect of temperature on grafting**			
50	18.60	12.82	81.76	48.40
55	46.02	31.72	100.66	59.59
60	61.86	42.64	111.58	66.05
65	62.06	42.78	111.72	66.13
70	42.02	28.97	97.90	57.95
**Time (min)**	**Effect of time on grafting**			
30	49.80	34.33	103.27	61.13
60	53.52	36.90	105.83	62.65
90	60.24	41.53	110.46	65.39
120	62.06	42.78	111.72	66.13
150	52.02	35.86	104.80	62.03
**APS concentration (mmol)**	**Effect of initiator concentration on grafting**			
0.02	18.46	12.73	81.66	48.34
0.04	13.40	9.24	78.17	46.27
0.06	30.46	21.00	89.94	53.24
0.07	44.16	30.44	99.38	58.83
0.09	62.06	42.78	111.72	66.13
0.11	58.40	40.26	109.20	64.64
**AMPS concentration (mmol)**	**Effect of monomer concentration on grafting**			
1.5	9.61	15.46	176.29	67.59
2.0	25.86	31.19	151.82	68.81
2.5	37.80	36.48	132.98	67.67
3.0	40.71	32.74	113.16	62.72
3.5	62.02	42.75	111.68	66.11
4.0	60.28	36.36	96.67	60.30

Table S2: %Grafting, %grafting efficiency, %conversion, and %yield of PC-g-SVBS graft copolymer prepared by CAN as initiator based on effect of various reaction conditions.

	**%Grafting**	**%Grafting Efficiency**	**%Conversion**	**%Yield**
**Temperature (°C)**	**Effect of temperature on grafting**			
50	31.86	28.83	119.30	62.63
55	72.46	65.56	156.04	81.92
60	84.22	76.20	166.68	87.50
65	82.60	74.73	165.21	86.74
70	82.02	74.21	164.69	86.46
**Time (min)**	**Effect of time on grafting**			
30	38.00	34.38	124.86	65.55
60	44.72	40.46	130.94	68.74
90	72.64	65.72	156.20	82.00
120	84.22	76.20	166.68	87.50
150	84.18	76.16	166.64	87.49
180	84.00	76.00	166.48	87.40
**CAN concentration (mmol)**	**Effect of initiator concentration on grafting**			
0.009	22.46	20.32	110.80	58.17
0.018	38.12	34.49	124.97	65.61
0.027	60.46	54.70	145.18	76.22
0.036	84.22	76.20	166.68	87.50
0.046	79.86	72.25	162.73	85.43
**SVBS concentration (mmol)**	**Effect of monomer concentration on grafting**			
1.5	41.71	75.48	256.43	91.27
2.0	46.42	63.00	198.71	84.30
2.5	56.62	61.47	170.05	81.53
3.0	84.22	76.20	166.68	87.50
3.5	84.38	65.44	142.99	80.53
4.0	83.78	56.80	124.65	74.26

Table-S3: Various kinetics model for 5FU release studies.

Sample code	Zero order		First order		Higuchi		Hixson-Crowell		Korsmey		
									pH-1.2		
	R	***K_0***	***R***	***K_1***	***R***	***K_h***	***R***	***K_{hc}***	***R***	***n***	***k***
PVA	0.85	5.12	0.723	4.03	0.823	1.75	0.731	3.35	0.943	8.21	5.32
PSASB	0.954	0.681	0.989	1.35	0.991	0.58	0.995	1.44	0.995	2.22	0.309
PSAAM	0.977	1.133	0.990	1.00	0.990	0.43	0.966	1.22	0.993	0.217	0.865
